# Neurological manifestations of oculodentodigital dysplasia: a Cx43 channelopathy of the central nervous system?

**DOI:** 10.3389/fphar.2013.00120

**Published:** 2013-09-26

**Authors:** Marijke De Bock, Marianne Kerrebrouck, Nan Wang, Luc Leybaert

**Affiliations:** Physiology Group, Department of Basic Medical Sciences, Ghent UniversityGhent, Belgium

**Keywords:** oculodentodigital dysplasia, Cx43, gap junction, hemichannel, astrocyte

## Abstract

The coordination of tissue function is mediated by gap junctions (GJs) that enable direct cell–cell transfer of metabolic and electric signals. GJs are formed by connexins of which Cx43 is most widespread in the human body. In the brain, Cx43 GJs are mostly found in astroglia where they coordinate the propagation of Ca^2+^ waves, spatial K^+^ buffering, and distribution of glucose. Beyond its role in direct intercellular communication, Cx43 also forms unapposed, non-junctional hemichannels in the plasma membrane of glial cells. These allow the passage of several neuro- and gliotransmitters that may, combined with downstream paracrine signaling, complement direct GJ communication among glial cells and sustain glial-neuronal signaling. Mutations in the *GJA1* gene encoding Cx43 have been identified in a rare, mostly autosomal dominant syndrome called oculodentodigital dysplasia (ODDD). ODDD patients display a pleiotropic phenotype reflected by eye, hand, teeth, and foot abnormalities, as well as craniofacial and bone malformations. Remarkably, neurological symptoms such as dysarthria, neurogenic bladder (manifested as urinary incontinence), spasticity or muscle weakness, ataxia, and epilepsy are other prominent features observed in ODDD patients. Over 10 mutations detected in patients diagnosed with neurological disorders are associated with altered functionality of Cx43 GJs/hemichannels, but the link between ODDD-related abnormal channel activities and neurologic phenotype is still elusive. Here, we present an overview on the nature of the mutants conveying structural and functional changes of Cx43 channels and discuss available evidence for aberrant Cx43 GJ and hemichannel function. In a final step, we examine the possibilities of how channel dysfunction may lead to some of the neurological manifestations of ODDD.

## Introduction: ODDD mutations, clinical manifestations and review focus

Oculodentodigital syndrome or oculodentodigital dysplasia (ODDD, OMIM #164200) is a mostly autosomal dominant disease caused by mutations in the *GJA1* gene which is located on chromosome 6 (q21-q23.2). ODDD is a rare disease (prevalence < 1/1,000,000^1^) and symptoms have been mostly described in Caucasian families; it is uncertain though whether this is a matter of clustering or of inconsistent screening in other populations. In the affected families, male and female patients are found in equal numbers while in sporadic forms of ODDD, females seem to be more susceptible (reviewed in Paznekas et al., 2009; Avshalumova et al., 2013).

*GJA1* encodes for one of the most abundant connexin (Cx) proteins, Cx43. Cxs are a family of transmembrane proteins with molecular weights (MW) varying from 26 to 60 kilodaltons (kDa) on which the current nomenclature is based (Cx43 has a MW of ~43 kDa). In vertebrates, Cxs are the building blocks of gap junction (GJ) channels, intercellular channels that connect the cytoplasm of two neighboring cells. A GJ channel consists of two hemichannels (HCs), each composed of six Cx proteins and delivered by each of the coupled cells. Cx43 is ubiquitously present in the human body in a large array of tissues and cells (reviewed in Laird, 2006). As a result, ODDD patients exhibit a pleiotropic phenotype with manifestations in a large variety of organ systems. Externally, mostly eyes, teeth, hands and feet are affected. Typical craniofacial dysmorphisms include a thin nose with hypoplastic *alae nasi*, small anteverted nares and prominent *columnella*. Some patients additionally have dysplastic ears. Ophthalmological anomalies include microphthalmia, microcornea, iris abnormalities, cataracts, glaucoma and optical neuropathy. Malformations of the extremities are another hallmark of ODDD and include syndactyly of fingers and toes. Additionally, campylodactyly (fixed flexion deformity of fingers and toes) and clinodactyly (lateral curvature of the fingers) are frequently observed. In the oral region, mandibular overgrowth, cleft lip, and cleft palate may be present. Abnormalities in primary and permanent teeth such as microdontia, partial anodontia, enamel hypoplasia, caries, and early tooth loss are observed in most patients. Brittle nails and hair abnormalities sometimes appear and skin diseases like palmoplantar keratoderma and subclinical wound healing defects are possible as well (Paznekas et al., 2003; Gong et al., 2006; Thibodeau et al., 2010; Churko et al., 2011; Amano et al., 2012).

In addition to this large variety of physically observable features, the disease is also characterized by cardiac and neurological malfunctioning. The cardiac phenotype is observed in 3% of the ODDD patients and includes endocardial cushion defects, atrial or ventricular septum defects, recurrent ventricular tachycardia, atrioventricular block, and idiopathic atrial fibrillation (Paznekas et al., 2003, 2009). Cardiac arrhythmia caused by a Cx43 mutation (E42K) has been associated with “sudden infant death syndrome” (Van Norstrand et al., 2012), making it plausible that some of the ODDD mutations result in a cardiac phenotype. Neurological symptoms are not universally seen, but about 30% of the patient population has been diagnosed with neurological problems that include conductive hearing loss, dysarthria (speech articulation problems), neurogenic bladder (voiding problems), ataxia (gait disturbance), muscle weakness, spasticity, and seizures. MRI imaging studies have brought up diffuse bilateral abnormalities in the subcortical cerebral white matter, possibly indicating a slow progressive leukodystrophy. Mental retardation may occur but is rare (Paznekas et al., 2003, 2009; Amador et al., 2008; Joss et al., 2008; Furuta et al., 2012). Of note, in most families, the appearance of neurological symptoms is unpredictable, but in 4 families genotyped for having the L90V, L113P, K134N, or G138R mutant, all ODDD patients exhibit neurological traits (Paznekas et al., 2009). Cardiac and neurological manifestations in ODDD have only started to emerge in the past decade and the list of “new” ODDD features still seems to expand continuously. Brice and co-workers have for instance recently described a case of lymphedema in ODDD (Brice et al., 2013). Given the low prevalence, the high pleiotropy and the still growing list of previously unnoticed ODDD features, currently, there are no clear data available about the life expectancy of the patients.

In this review, we focus on the neurological manifestations of ODDD and explore how Cx43 mutations and consequent channel aberrations may link to some of these manifestations (Table 1). We start with an overview of connexins and its channels (section Connexin Channels: Gap Junctions and Hemichannels), provide an in depth analysis of the consequence of ODDD mutations on channel function (section ODDD-Linked Mutations and Cx43 Channel Function) and end by examining how channel dysfunction may lead to alterations in neural tissue functioning (section Neurological Phenotype in ODDD—Link to Aberrant Cx43 Channels). Several excellent reviews have highlighted each of these aspects (Kielian, 2008; Laird, 2008, 2010; Chew et al., 2010; Orellana et al., 2011; Abrams and Scherer, 2012; Eugenin et al., 2012); here we aimed to correlate channel dysfunction with neurological manifestations and explore possible relations in the framework of currently available knowledge.

**Table 1:**
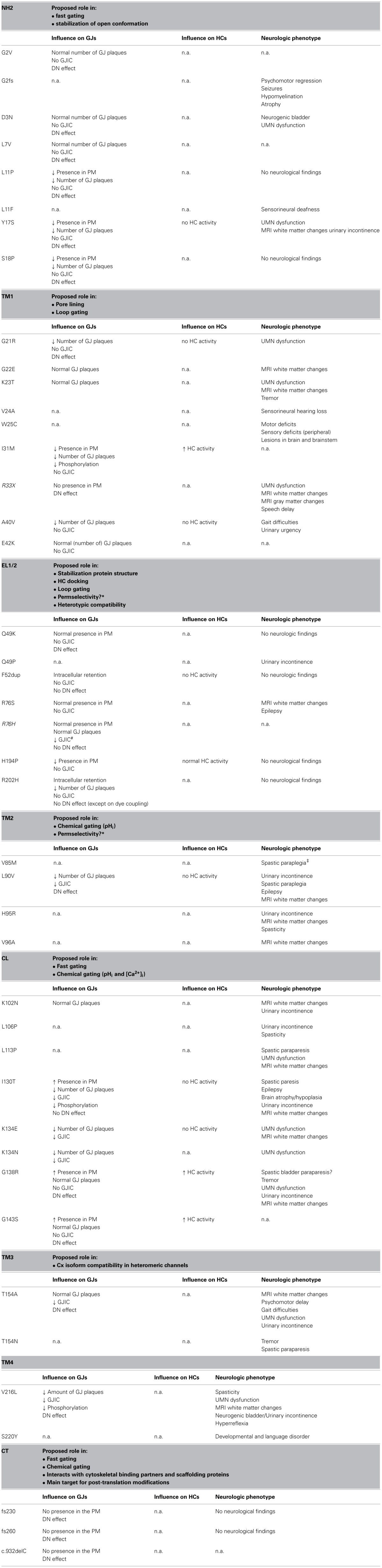
**Overview of ODDD-linked mutations in different Cx43 domains, their effect on Cx channel properties and the associated neurologic phenotypes**.

*For references see text. Neurological traits are from Paznekas et al. (2003) and Abrams and Scherer (2012) and references therein, unless indicated*.

^*^The role of the EL domains in the determination of Cx channel permselectivity has been suggested based on observations in Cx46 channels. TM2 is implicated in regulating permselectivity of Cx26 channels. Thus far, nothing is known on the role of these domains in regulating permselectivity of Cx43 channels.

*^#^Only for certain exogenous markers. With other markers, GJIC is similar to WT Cx43*.

^‡^Quintáns et al., 2013

*DN, dominant negative effect; n.a., data not available; PM, plasma membrane; UMN, upper motor neuron*.

*Mutants in italic font are inherited in an autosomal recessive manner*.

## Connexin channels: gap junctions and hemichannels

Cxs form two kinds of functional channels: GJs and HCs. GJs mediate the direct diffusion of ions and molecules with MWs up to 1.5 kDa, including inositol 1,4,5 trisphosphate (IP_3_), cyclic nucleotides, and energy molecules such as ATP (reviewed in Alexander and Goldberg, 2003), thereby contributing to the coordination of cell function in several organs and tissues. GJ channels are for instance implicated in the propagation of intercellular Ca^2+^ waves (ICWs) (reviewed by Leybaert and Sanderson, 2012), metabolic and electric coupling in astrocytes and cardiomyocytes (Rouach et al., 2008; Meme et al., 2009; Desplantez et al., 2012), exchange of bone modulating molecules (reviewed in Batra et al., 2012) and synchronization of smooth muscle cell contraction in the bladder and uterus (Miyoshi et al., 1996; Neuhaus et al., 2002). Moreover, GJs may also spread cell death signals to neighboring cells, thereby contributing to tissue/organ damage in pathology (Decrock et al., 2012). HCs can be present both as GJ precursors in the plasma membrane (PM) or as non-junctional channels, not incorporated into GJs. For a long time, it was thought that HCs remain closed until they form a GJ channel, since uncontrolled HC opening would lead to membrane depolarization and depletion of essential molecules from the cytoplasm, ultimately leading to cell dysfunction and possibly cell death. The first evidence of functional HCs, derived from *in vitro* work whereby Cx46 was expressed in *Xenopus laevis* oocytes, confirmed that HC opening resulted in uptake of Lucifer yellow, but also in depolarization and cell death (Paul et al., 1991). Research over the past decades has identified numerous scenarios in which HCs are activated (see section ODDD-Linked Mutations and Cx43 Channel Function). HCs have been shown to be involved in different forms of paracrine signaling through the release of ATP (Kang et al., 2008), glutamate (Ye et al., 2003), glutathione (Rana and Dringen, 2007), NAD^+^ (Goodenough and Paul, 2003), and prostaglandins (Jiang and Cherian, 2003; Orellana et al., 2011). HC-mediated ATP release functions as a paracrine signal in the propagation of ICWs (Leybaert and Sanderson, 2012) and evidence is accruing that HCs may additionally contribute to “center-surround” antagonism in the retina (Kamermans et al., 2001; Goodenough and Paul, 2003), osteogenesis (reviewed in Civitelli, 2008; Batra et al., 2012), regulation of vascular permeability (De Bock et al., 2011), central chemoreception (Huckstepp et al., 2010), atherosclerotic plaque formation (Wong et al., 2006), induction of astrogliosis (O'Carroll et al., 2008), ischemia-related cell death (Danesh-Meyer et al., 2008, 2012; Davidson et al., 2012; Wang et al., 2013a,b and reviewed in Contreras et al., 2004; Bargiotas et al., 2009) as well as in the propagation of apoptotic signals (Decrock et al., 2009). The role of HCs has been heavily debated since the discovery of pannexin channels, transmembrane channels that have similar tissue distribution and properties as HCs but are not likely to form GJ channels (Spray et al., 2006; Iglesias et al., 2009; Scemes, 2012). Much of the published data on the possible role of both HCs and pannexin channels is based on indirect measures that might be prone to misinterpretation and these issues are considered in detail in another review in this Frontiers Research Topic (Giaume et al., 2013).

### Connexin life cycle and channel assembly

Because of the relatively short Cx half-life (1–6 h), there is a continuous synthesis and breakdown of the protein, enabling fast adaptation of GJ intercellular communication (GJIC) to the physiological needs of the tissue (reviewed in Herve et al., 2007; Rackauskas et al., 2010). This is for instance illustrated in the myometrium, where steroid hormones control the expression level of Cxs before and after parturition (Risek et al., 1990). Like most transmembrane proteins, Cxs are co-translationally integrated into the rough endoplasmic reticulum (ER) membrane where they adopt their native transmembrane configuration (Falk, 2000; Vanslyke et al., 2009). Hydropathy plots reveal that all Cx proteins share a common topology: four transmembrane alpha-helices (TM1-4) are connected through two extracellular loops (EL1-2). In each loop, three cysteine residues form intramolecular disulphide bonds that are required for appropriate folding of the protein (Dahl et al., 1992; Foote et al., 1998). The Cx protein further contains three intracellular domains: a cytoplasmic loop (CL), an amino-terminal domain (NT), and the carboxyl-terminal region (CT). The best conserved protein regions are the ELs and the TM domains, whereas the CT- and CL-regions show marked divergence (reviewed in Nicholson, 2003; Saez et al., 2003; Wei et al., 2004). The subsequent oligomerization of Cx proteins into HCs starts in the ER, progressing to the trans-Golgi network (Falk, 2000; Vanslyke et al., 2009). During this process, newly synthesized HCs remain closed in order to protect and maintain the integrity of the lumens of the intracellular compartments (Moreno, 2005; Laird, 2006).

After leaving the ER, Cxs first pass the ER-Golgi intermediate compartment and then transit through the cis- and trans-Golgi network before being shuttled to the PM. Pleomorphic vesicles and tubular extensions departing from the Golgi apparatus contribute to the delivery of HCs to the PM (Gaietta et al., 2002; Laird, 2006). It is not entirely clear to what extent post-translational modifications are required for Cx transport to the PM. Some data suggest that Cx43 is transiently phosphorylated early in the secretory pathway, but the vast majority of phosphorylation is thought to occur at the cell surface (Lampe et al., 2000; Solan and Lampe, 2007 and reviewed in Lampe and Lau, 2004), where it plays a complex role in Cx channel gating (see further). Notably, once inserted into the cell membrane, HCs undergo specific adhesions and dock with HCs of neighboring cells to form GJs. Hundreds to thousands of GJ channels cluster in plaques at the cell–cell interface with newly formed GJs located at the border of the plaque and older channels located centrally. These central channels are targeted for internalization and degradation of GJ channels (reviewed in Goodenough and Paul, 2003; Laird, 2006).

Internalization is mediated through large double-membrane bound vesicles that contain a complete GJ channel. These vesicles are called “annular junctions” or “connexosomes” (Gaietta et al., 2002; Laird, 2006) and also contain Cx-binding proteins and molecules that function as chaperones for internalization and intracellular degradation through the proteasomal or lysosomal pathway (Qin et al., 2003; Thevenin et al., 2013). Cxs have been shown to be a substrate for ubiquitination that is known to guide proteins to proteasomes. Connexosomes are identified in the majority of cell types, but are for instance difficult to find in hepatocytes. It therefore remains possible that Cxs are additionally internalized and degraded through the endosomal cascade. Furthermore, internalization of Cx43 has been suggested to occur via a clathrin-mediated, caveolae-dependent process. Clearly, more studies are required to investigate all possible pathways of internalization/degradation. The proteasomal pathway may be responsible for ER-associated degradation, while lysosomes degrade Cxs that are recycled to the PM trough endosomes (Laird, 2006).

## ODDD-linked mutations and Cx43 channel function

### Cx43 mutations and channel gating

Collectively, there are over 62, mostly missense, mutations found throughout the expanse of the Cx43 protein that are associated with ODDD. All Cx domains have specific functions, be it in Cx trafficking, Cx assembly or channel gating. In contrast to “benign” polymorphisms that have no discernible effect on Cx43 channel function, a minority of mutants promote channel opening/function; however, most mutants carry loss-of-function mutations and may involve those leading to inappropriate membrane sorting, those leading to improper folding, interfering with the HC docking process, and those giving rise to altered permselectivity or gating. The latter implies a fast and reversible shift in the channel's conductive properties which can be due to conformational changes or which can be mediated by Cx-linked adaptor molecules and proteins (Bukauskas et al., 2000; Cottrell et al., 2003; Herve et al., 2007; Moreno and Lau, 2007).

Cx channels—both GJs and HCs—are sensitive to changes in voltage, intracellular pH and [Ca^2+^]_*i*_, allowing swift adaptations (faster than those brought about by assembly/disassembly) of GJIC and paracrine signaling to comply to the specific needs of the tissue. In terms of voltage-dependent channel gating, GJs are influenced mainly by the transjunctional voltage (*V*_*j*_) which defines the difference in voltage measured between two coupled cells. In contrast, HCs are highly sensitive to *V*_*m*_: they remain preferentially “silent” at inside negative potentials but are activated upon depolarization by a gating mechanism that resembles gating transitions associated with the docking of extracellular loop domains; therefore this gating is also referred to as loop gating (or slow gating) (Trexler et al., 1996; Bukauskas and Verselis, 2004; Gonzalez et al., 2006; Verselis et al., 2009). Loop gating represents transitions between the fully open and closed states whereas fast (<1 ms) HC gating involves transitions to long-lasting substates. Chemical gating, achieved by changes in the intra- or extracellular environment, much resembles gating transitions observed with slow voltage gating; therefore, it has been proposed that both phenomena are mediated by the same mechanisms.

Cx43 mutations are found in all protein domains, each of which has been associated with specific functions in terms of channel oligomerization, trafficking, gating and permeability. Importantly, many of the known mutations causing ODDD occur in residues that are highly conserved throughout the animal kingdom (Paznekas et al., 2003), hinting toward their importance in channel regulation. Below, we discuss the different Cx43 mutations that have been characterized in terms of trafficking, channel assembly and channel function. Unfortunately though, for most mutant channels there is not yet a clear structural-functional correlation that would allow a better understanding of what is going wrong with the channels. The effects of the Cx43 mutations are often not predictable from their location in the primary structure and mutations located at different domains may cause similar channel dysfunction phenotypes, ultimately leading to the ODDD phenotype.

### N-terminal Cx43 mutations

Deletion of the first N-terminal amino acids (AAs 2–7) disables Cx43 trafficking and most of the Cx43 protein is retained in the intracellular compartment. Overall, the Y17S mutation has similar effects (Shao et al., 2012), although a few plaque-like structures remained observable at the PM (Shibayama et al., 2005; Lai et al., 2006). Nevertheless, given the problematic trafficking of this mutant, neither HC activity nor GJIC was detected (Lai et al., 2006). Unfortunately, fairly little is known on the role of the Cx43 N-terminal domain in terms of oligomerization or trafficking. Most of the other N-terminal missense mutations, including G2V, D3N, L7V, L11P, and S18P, do allow a normal Cx life cycle, but result in non-functional GJs, as evidenced by the lack of dye transfer and the absence of electrical coupling (Shibayama et al., 2005; Churko et al., 2011; Shao et al., 2012). In these mutant channels, the closed state is thus relatively more stable than the open one. Importantly, these mutant proteins exert a dominant negative effect on WT Cx43 in terms of coupling, indicating that the patients are likely experiencing more than 50% loss of normal Cx43 function, despite having one functional allele (Shao et al., 2012).

A hydrophobic core built around W4 has been put forward as a structural determinant of the Cx43 N-terminal domain, governing interactions with the TM1 domain. Replacement of G2 with a valine residue was suggested to introduce additional hydrophobic interactions, not present in the WT channel that would abolish NT-TM1 interactions (Shao et al., 2012). Based on electron crystallography of Cx26 channels and electrophysiological studies performed in Cx46 channels, interactions between the NT and the TM1 domains are suggested to keep the channel in the open conformation (Maeda et al., 2009; Kronengold et al., 2012). Preventing NT-TM1 interactions may thus be one mechanism by which the mutants result in non-functional GJ channels.

### Cx43 mutations in the transmembrane domains

The crystal structure of a C-terminally truncated Cx43 channel at 7.5 Å resolution indicates the presence of 24 transmembrane α-helices within each hemichannel, grouped in two concentric rings around the central pore (Unger et al., 1997, 1999); however, there has been controversy regarding the identities of the principal pore-lining segments. The substituted cysteine accessibility method (SCAM) that enables the identification of pore lining residues, has pointed out that TM3 might delineate the inner pore of Cx32 GJs (Skerrett et al., 2002); however, currently, most evidence points to the amphipathic TM1 as the major pore-lining domain in channels composed of Cx46 (Zhou et al., 1997; Kronengold et al., 2003a,b), Cx50 (Verselis et al., 2009) Cx32 (Oh et al., 1997; Tang et al., 2009) and Cx26 (Sanchez et al., 2010). The crystal structure of a Cx26 channel at 3.5 Å resolution confirms that TM1, but also TM2, are part of the inner lumen wall, whereas TM3 and TM4 face the hydrophobic environment (Maeda et al., 2009).

In TM1, mutants G21R, G22E, K23T, I31M, R33X, A40V, and E42K have all been identified in ODDD patients. Of these G21R, G22E, K23T, and E42K [the latter causing sudden infant death syndrome (Van Norstrand et al., 2012)], encounter normal trafficking to the PM and insertion into GJ plaques; yet cells are not electrically coupled (data on channel function of G22E and K23T mutant channels are still lacking) (Roscoe et al., 2005; Shibayama et al., 2005; Abrams and Scherer, 2012; Van Norstrand et al., 2012; Huang et al., 2013). When the Cx43-G21R mutant is co-expressed with endogenous WT Cx43, it negatively influences dye coupling (Shibayama et al., 2005). Both I31M and A40V mutants exhibit disturbed membrane insertion with just a fraction of the channels present in GJ plaques; these were, however, not sufficient to sustain GJIC (Shibayama et al., 2005; Dobrowolski et al., 2007). With respect to HC activity, G21R and A40V do not form functional HCs but I31M mutant channels release more ATP compared to the Cx43 WT. The half-life of the I31M mutant was not prolonged pointing to altered gating mechanisms or a shift in permselectivity (Lai et al., 2006; Dobrowolski et al., 2007). Additionally, Cx43-I31M presents with reduced phosphorylation that is, up to a certain degree, required for proper GJIC (Dobrowolski et al., 2007). This is quite surprising as Cx43 has thus far not been shown to be phosphorylated in transmembrane domains (reviewed in Johnstone et al., 2012; Marquez-Rosado et al., 2012). In addition, isoleucine residues are not generally direct targets for phosphorylation. Thus, it is more likely that a phosphorylation in TM1 is associated with conformational changes that somehow mask candidate phosphorylation sites in other protein domains.

Known mutations located in TM2 (L90V), TM3 (T154A), or TM4 (V216L) all form channels that are present as punctae in the PM, but the coupling capacity of each mutant channel is reduced. All mutants furthermore exert dominant negative effects on WT Cx43 when in a heteromeric or heterotypic conformation (McLachlan et al., 2005; Shibayama et al., 2005; Beahm et al., 2006; Churko et al., 2011). The H95R mutant (TM2) has additionally been described in an ODDD patient, but unfortunately, the manuscript provided no functional data (Honkaniemi et al., 2005). Earlier data indicate that this residue is involved in pH sensitivity of Cx43 channels (Ek et al., 1994): introducing a negative or positive charge at position 95 yields GJ channels that exhibit reduced or increased sensitivity to intracellular acidification, respectively. Finally, residues located in TM2 have been proposed to play a role in determining permselectivity. Permeation of the Ca^2+^ mobilizing messenger IP_3_ for instance, is abolished in GJ channels composed of the mutant Cx26-V84L which is associated with hereditary deafness. The mutant protein has, however, no effect on unitary conductance and permeability to Lucifer yellow, which remain indistinguishable from WT Cx26 (Beltramello et al., 2005). This suggests that subtle structural modifications in the channel pore may selectively hinder the passage of biologically relevant molecules, possibly by altering certain interactions between the permeant and the channel pore. As this valine is highly conserved (V85 in Cx43) it may well be involved in determining IP3 transfer through Cx43 channels. ODDD patients carrying a V85M mutant were recently identified (Fenwick et al., 2008; Quintáns et al., 2013), but again, the implications of this mutant on channel trafficking/gating/permselectivity were not studied.

### Cx43 mutations in the extracellular loops

The extracellular loops are characterized by conserved, cysteine-rich patterns (CX_6_CX_3_C in EL1 and CX_4/5_CX_5_C in EL2), and intramolecular disulphide bridges in and between the ELs stabilize the protein's tertiary structure (Dahl et al., 1992; Bruzzone et al., 1996; Foote et al., 1998). In Cx43 these involve C54, C61 and C65 in EL1 and C187, C192 and C198 in EL2. Mutations of each of these cysteines could result in distorted loops resulting in improper protein folding and retention in intracellular compartments. Although mutations of conserved cysteines have not been associated with ODDD, two mutations that lie in direct proximity to conserved cysteine residues (F52dup and Cx43-R202H) cannot be traced back to the PM (Shibayama et al., 2005; Lai et al., 2006). Other mutants (Q49K, R76S/H, H194P) are inserted into the lipid bilayer (Sokolova et al., 2002; McLachlan et al., 2005; Shibayama et al., 2005; Dobrowolski et al., 2007; Abrams and Scherer, 2012; Huang et al., 2013); yet, electrical coupling is abolished (Sokolova et al., 2002; McLachlan et al., 2005). Based on a model proposed by Foote et al. in which the interdigitation of apposed ELs is required for docking and thus GJ formation (Foote et al., 1998), even those mutants that are present in the PM, may not form GJs due to distorted loops. This is further supported by the fact that HC activity of Cys-less Cx43 mutant channels appears normal both *in vitro* and *in vivo* (Bao et al., 2004; Tong et al., 2007). Also for ODDD-linked mutants like H194P in which the insertion of a proline induces a kink in the EL2 domain, HC activity is normal (Dobrowolski et al., 2007). In addition, those mutant proteins that cannot traffic to the PM in a homomeric configuration, can do so when assembled in a heteromeric channel with WT Cx43 (Shibayama et al., 2005; Lai et al., 2006). Interestingly, main and subconductance states of these WT:R202H and WT:F52dup channels were not different from homomeric WT channels (Shibayama et al., 2005). The single channel conductance of Cx43-R76H channels was substantially reduced compared to WT Cx43 channels (Huang et al., 2013). Slow voltage gating of Cx channels generally involves the coordinated response of all Cx monomers in the channel (Bukauskas and Verselis, 2004; Kwon et al., 2012). This might explain why a remaining electrical coupling is observed in heteromeric WT:R202H channels. At the same time, however, the mutant proteins may narrow the pore as compared to homomeric WT channels, preventing the diffusion of larger molecules like Lucifer yellow while leaving its electrical conduction intact (Shibayama et al., 2005).

### Cx43 mutations located in the cytoplasmic loop

It is now firmly established that the CL domain is of utmost importance for Cx channel gating. It has for instance been shown to contain the preferred interaction site for calmodulin (CaM; region 136–158) and as such, to mediate the closure of GJ channels in response to increasing [Ca^2+^]_*i*_ (Zhou et al., 2007; Myllykoski et al., 2009). Indeed, Ca^2+^-induced closure of GJs is likely to be mediated by intracellular effector proteins since the uncoupled state may persist long after [Ca^2+^]_*i*_ has been restored (Cotrina et al., 1998; Lurtz and Louis, 2003, 2007). Even more important, it is now widely accepted that the L2 region (second half of the CL; amino acids 119–144) serves as a receptor domain for the CT and that this CT-CL interaction, also known as the ball-and-chain mechanism, is implicated in both voltage gating and chemical gating of GJs by intracellular pH changes (Ek et al., 1994; Ek-Vitorin et al., 1996; Bukauskas et al., 2000; Anumonwo et al., 2001; Moreno et al., 2002; Shibayama et al., 2006). A CT-CL interaction is also important for the regulation of Cx43 HCs by extracellular Ca^2+^, since a conformational change induced by an increase of extracellular Ca^2+^ masks the CT from antibody binding, which is most likely to be the result of its association with the “receptor”-domain (Liu et al., 2006). The modulation of HCs in response to changes in [Ca^2+^]_*i*_ has only been outlined over the last decade, demonstrating that HCs are differently influenced by [Ca^2+^]_*i*_ as compared to GJs. While GJs generally close with a [Ca^2+^]_*i*_ elevation, HCs display a bimodal response: a moderate increase in [Ca^2+^]_*i*_ up to 500 nM strongly promotes HC opening while this effect disappears with [Ca^2+^]_*i*_ > 500 nM. Further increases in [Ca^2+^]_*i*_ to the micromolar level tend to close the HCs (Shintani-Ishida et al., 2007; De Vuyst et al., 2009; Wang et al., 2012a). Mechanistically, Ca^2+^-activation of Cx43 HCs is mediated by CaM-dependent signaling (De Vuyst et al., 2009) and necessitates a CT-CL interaction (Ponsaerts et al., 2010). Most notably, the necessity of CT-CL interaction to trigger opening of Cx43 HCs stands in stark contrast to the fact that such interaction results in closure of GJs (reviewed in Iyyathurai et al., 2013). Addition of a CT-mimicking peptide prevented HC closure at high [Ca^2+^]_*i*_ and restored HC activity of CT truncated Cx43 (Ponsaerts et al., 2010), while not affecting HC activation by modest (<500 nM) [Ca^2+^]_*i*_.

Several ODDD-associated mutations have been identified in the CL domain, most of which are located in the L2 region. Cx43-I130T HCs are transported to the PM and assemble into GJ plaques. Moreover, mutant levels in the PM exceed those of WT Cx43 which might indicate a reduced degree of protein degradation. Despite of this, the mutant exhibits reduced dye and electrical coupling and HC activity is disrupted (Shibayama et al., 2005; Lai et al., 2006; Kalcheva et al., 2007). K134E/N mutants show a reduced degree of plaque formation and electrical coupling and a decrease in unitary conductance (Shibayama et al., 2005). Both I130 and K134, forming ionic bonds with C-terminal aspartate residues, are suggested to be involved in CT-CL interactions (Seki et al., 2004; Hirst-Jensen et al., 2007). As discussed higher, CT-CL interactions are necessary for HC opening while they result in closure of GJs. However, I130T and K134E mutant channels are closed in both the GJ and HC configuration, indicating that mechanisms different from altered CT-CL interactions may contribute to the closure of GJs.

Two CL mutations located further downstream in the L2 region (G138R and G143S), result in GJ channels that fail to sustain electrical or dye coupling and have a dominant negative effect on WT or endogenous (NRK—normal rat kidney—cells) Cx43. Interestingly, HC activity, measured by dye uptake and ATP release, is increased in both mutants (Roscoe et al., 2005; Dobrowolski et al., 2007, 2008). It is possible that the differential effects of these mutants on GJs and HCs mutants result from reinforced CT-CL interactions, caused by stronger electrostatic interactions between the CL equipped with an additional positively charged arginine residue (G138R), with negatively charged residues in the CT (Gong et al., 2007). Likewise, a shift from a non-polar glycine to a polar serine residue (G143S) may account for a higher number of hydrogen bonds that lock the CT to the CL. Further work is needed to substantiate these possibilities.

### C-terminal Cx43 mutations

As highlighted above, the CT functions as a gating particle that alters its state in response to changes in the extra-or intracellular environment. The CT domain is additionally the primary target for post-translational modifications like S-nitrosylation (Retamal et al., 2006) and phosphorylation (reviewed by Johnstone et al., 2012). The majority of phosphorylation events occur on serine residues although tyrosine phosphorylation is abundant as well (Lampe and Lau, 2004; Solan and Lampe, 2007). Phosphorylation of Cx proteins seems to be intricately involved in Cx trafficking to the PM. Additionally, both under basal and stimulated conditions, Cx channel activity appears to be regulated by ongoing phosphorylation-dephosphorylation events. Substitution of C-terminal serines (S325, S328, S330) by alanines has indicated that phosphorylation of these serines is required for a fully open state of Cx43 GJs while phosphorylation of other residues (S255, S279, S282, S368, Y247, and Y265) favors channel closure (Ek-Vitorin and Burt, 2013). However, much of the details on how Cx phosphorylation can determine trafficking, turnover and the activity state of HCs and GJs still remains to be resolved (Johnstone et al., 2012). One detailed example of Cx channel regulation by phosphorylation is phosphorylation of the Cx43 CT tail by pH-dependent kinases which might introduce negative charges at this site, potentiating CT-CL interaction (Yahuaca et al., 2000).

Although the CT is the primary interaction domain of Cx-associated partner proteins like *zonula occludens* 1, tubulin, microtubules, and caveolins that may regulate protein trafficking and function (Giepmans et al., 2001; Langlois et al., 2008; Saidi Brikci-Nigassa et al., 2012); work with CT-truncated Cx43 mutants has repetitively shown that CT-truncated proteins are present at the PM of mammalian cells (Unger et al., 1999; Moreno et al., 2002; Kang et al., 2006; Maass et al., 2007). Oppositely, most of the ODDD-linked CT mutations are not inserted in the PM: the frame shift mutations fs230 and fs260 result in preliminary truncated Cx43 and present with reduced ability to form plaques while having a dominant negative effect on WT Cxs with respect to trafficking (Lai et al., 2006; Gong et al., 2007; Churko et al., 2011). In the atrial tissue of a patient with idiopathic atrial fibrillation, a frame shift mutation caused by a single nucleotide deletion (c.932delC) was identified. The mutation renders a Cx43 protein exhibiting aberrant CT amino acids starting from position 346 followed by a premature stop codon, leading to prompt truncation. The protein remains intracellular and exerts a dominant negative effect on wild type Cx43 (as well as Cx40) in the atrial tissue (Thibodeau et al., 2010). Importantly, all previously reported work with CT-truncated mutants was indeed based on exogenous expression systems and the c.932delC mutant was similarly observed at the PM when exogenously expressed in HeLa cells where it even sustained dye coupling, though to a smaller extent than WT Cx43 (Hong et al., 2010). Another major difference between exogenous CT-truncated mutants and the ODDD mutants is that in the former the CT is absent while with frame shifts and single nucleotide deletion mutants, a CT is still (partly) present, albeit with a wrong amino acid sequence which could give a different outcome on trafficking.

## Neurological phenotype in ODDD - link to aberrant Cx43 channels

Apart from the physical appearances linked to ODDD, several of the above described mutations have been associated with seizures, spasticity, gait difficulties, tremor and incontinence. These symptoms are for the bigger part clinical manifestations of underlying neuronal damage in the central nervous system (CNS). Patients may also suffer from hearing loss and decreased visual acuity or blindness; however, the latter two phenotypes are infrequent and only observed in a small fraction of the patients presenting with neurological traits. Magnetic resonance imaging (MRI) and computed tomography (CT) scans of patients' brains revealed abnormalities in both white and gray matter (Loddenkemper et al., 2002; Amador et al., 2008; Joss et al., 2008; Paznekas et al., 2009; Alao et al., 2010; Abrams and Scherer, 2012). One patient was reported to exhibit central or sensorineural hearing loss which is associated with damaged cranial nerve VIII (cochlear part) that transmits signals from the cochlea to the inner ear. Loss of visual acuity (decreased sharp vision) or blindness result from atrophy of the optic nerve that relays visual information from the retina (via the thalamus) to the visual cortex. Similar loss of visual acuity follows optical nerve neuritis observed in the demyelinating disease multiple sclerosis (Florio and Maniscalco, 2011). Spasticity is a muscle control disorder characterized by stiff, uncontrollable muscles with hyperreactivity toward exogenous stimulation, and is generally associated with hyperactive, persisting reflexes (hyperreflexia). Tremor is characterized by involuntary rhythmic muscle contractions. According to the alpha/gamma-coactivation theory, muscle contraction is controlled by both alpha and gamma motor neurons. The alpha component delivers a feed forward command resulting in force delivery by activating extrafusal muscle fibers. The gamma motor component controls the length of muscle spindles (intrafusal muscle fibers), thereby delivering input to a servo-controlled neuronal circuit that receives feedback on the length status from the sensory output of muscle spindles. Spasticity may develop as a result of aberrantly increased gamma motor neuron activity, resulting in increased tension in the muscle spindles that become oversensitive to external stimulation. Increased gamma motor drive may occur as a consequence of decreased central (cerebral and cerebellar) inhibitory input on spinal gamma motor neurons (reviewed in Sheean and McGuire, 2009). Ataxia is a cerebellar phenomenon that manifests as a lack of voluntary muscle coordination, typically observed as disturbances in the gait pattern. Interestingly, one patient carrying the W25C (TM1) mutation presented with all characteristic craniofacial features and extremity anomalies, as well as with spastic tetraparesis, hyperreflexia and sensory disturbances (numbness in feet), indicating wide-spread sensori-motor neurological deficits (Furuta et al., 2012). In two siblings characterized for having an R33X mutation, myelination deficits were described (Joss et al., 2008). The other major neurological deficit, namely seizure activity, results from a repetitive and synchronized, excessive neuronal activity in the CNS. Epilepsy has long been viewed as a channelopathy and involves epileptogenic (genesis of the disease) and ictogenic (genesis of an epileptic ictus or seizure) components. Currently, our understanding of epileptogenesis is very incomplete, at least at a mechanistic level. Ictogenesis is related to an imbalance between glutamatergic excitation and (mostly) GABA-ergic inhibition, and has been associated with dysfunctional channels, mostly voltage-gated Na^+^, K^+^, Cl^−^, and Ca^2+^ channels that determine neuronal action potential firing. Moreover, the extracellular concentration of Na^+^, K^+^, Cl^−^, and Ca^2+^ may influence the glial cells and thereby exert control over neuronal activity. It is hypothesized that dysfunctional Na^+^, K^+^, Cl^−^, or Ca^2+^ channels alter the threshold for neuronal depolarization and action potential firing, thereby shifting the balance between excitation and inhibition. At the microscopic level, epileptic brain regions are characterized by injured neurons, gliosis, axonal sprouting, and the formation of new, aberrant, synaptic connections (reviewed in D'Ambrosio, 2004; Dichter, 2009; Reid et al., 2009).

**Figure 1:**
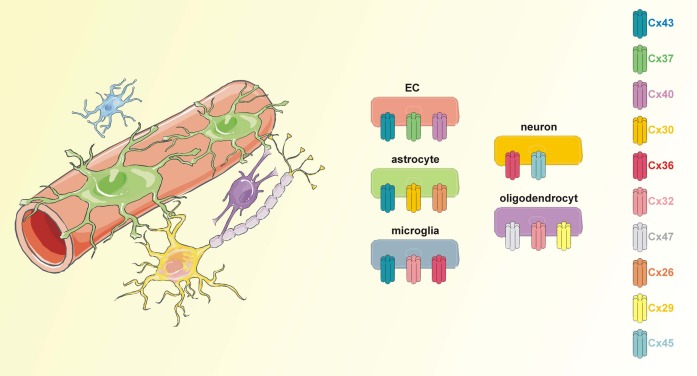
**Connexins in the neurogliovascular unit**. The neurogliovascular unit is a functional unit in which astrocytes are centrally involved. Astrocytic endfeet are tightly associated with endothelial cells that form the BBB while at their other site, astrocytes contact pre- and postsynaptic neuronal membranes. Additionally, astrocytes may contact other glial cells like microglia and oligodendrocytes. Each of the cell types in the neurogliovascular unit is endowed with a set of connexins proteins. Importantly, Cx43 is present in astrocytes as well as in BBB endothelial cells and microglial cells.

Cx43 is abundant in astrocytes and can additionally be found in activated microglia, developing neurons, and endothelial cells (Orellana et al., 2009, 2011; Avila et al., 2011; Wang et al., 2012b) (Figure 1). Given its ubiquitous presence, it is not surprising that around 30% of the ODDD patients exhibit neurological symptoms. This number may further increase as neurological investigations of ODDD patients are becoming progressively more detailed.

### ODDD-associated mutations and their implications for astrocyte functioning in the neurogliovascular unit

Despite being originally described as “brain glue,” established functions of glia nowadays suggest an active role in brain function and information processing (reviewed in Allen and Barres, 2009). Together with neurons, glial cells work in concert with vascular and blood cells to establish the neurovascular unit, which should better be called the neurogliovascular unit (Figure 1). The concept of a neurogliovascular unit has been central in exploring improved strategies and approaches to treat stroke patients (reviewed in Berezowski et al., 2012; Kur et al., 2012).

Astrocytes are involved in key aspects of the nervous tissue including preservation of blood-brain barrier (BBB) integrity, modulation of blood supply, maintenance of brain homeostasis, myelination, and neurotransmission. The combined ablation of Cx30 and Cx43, which results in coupling-deficient astrocytes, lowers the threshold for epileptiform events (Theis et al., 2003; Wallraff et al., 2006), alters astrocyte energy metabolism (Rouach et al., 2008), gives rise to swollen astrocytic endfeet (Ezan et al., 2012) and leads to parenchymal vacuolation (Lutz et al., 2012). Astrocyte-targeted deletion of Cx43 severely reduces cell–cell coupling although it is not completely abolished, likely due to compensatory actions of Cx30 (Theis et al., 2003; Wallraff et al., 2006; Unger et al., 2012). Astrocyte-specific Cx43-ablated mice have been shown to exhibit reduced motor performance (Frisch et al., 2003), similar to ODDD patients suffering from cerebellar ataxia; yet, astrocyte-targeted deletion of Cx43 does not affect viability or astrocyte morphology nor does it cause neurodegeneration or astrogliosis (Theis et al., 2003). Conclusions from knock-out studies should be drawn with caution, as recent work in the heart has indicated that transgenic animals with a 5 amino acid deletion of the Cx43 CT have a much stronger cardiac phenotype than those with a complete deletion of the CT (Lubkemeier et al., 2013). In addition, mice missing one copy of the Cx43 allele do not mimic ODDD (Paznekas et al., 2009).

Below we describe how aberrant astrocytic signaling, due to defective Cx43, may contribute to ODDD-linked neurological symptoms.

#### Connexins and astrocytes contribute to blood-brain barrier function

Proper electrical signaling in the CNS requires a strict composition of the microenvironment around synapses and axons. The composition of the brain interstitial fluid is largely maintained by capillary endothelial cells that constitute the blood-brain barrier (BBB) and that survey solute diffusion in and out the brain (a highly comparable barrier is present between the capillaries and nervous tissue in the spinal cord). Astrocytic endfeet nearly completely enwrap these capillary endothelial cells, inducing and maintaining barrier properties. Cx43 may contribute to astrocyte maintenance of the endothelial barrier, as mice lacking Cx30 and astroglial Cx43 have a weakened BBB that is more vulnerable to hydrostatic vascular pressure and shear stress but that is otherwise intact in the absence of a pathological insult (Ezan et al., 2012; Lutz et al., 2012). It is not known whether this is mediated by the Cx protein itself or by an effect related to GJs or HCs. It is equally unknown whether a putative GJ involvement would be limited to GJs between astrocytic endfeet or between endfeet and endothelial cells. *In vitro* evidence has suggested astrocyte-endothelial communication via GJs (as well as HCs) (reviewed in Braet et al., 2001), but *in vivo* evidence argues against this possibility (Simard et al., 2003).

In a first approximation, it is conceivable that Cx43 mutations that result in a trafficking defect or loss of channel function may give similar manifestations as Cx43 silencing. BBB leakage enables the entry of potentially neurotoxic, circulating compounds that directly affect neuronal survival, but also gives rise to inflammation, edema and hypoxia, which will ultimately result in additional injury to the neural tissue. At the same time, the ionic homeostasis of the brain's interstitial compartment becomes disturbed, particularly the increased K^+^ concentration will interfere with action potential propagation and synaptic transmission. Additionally, influx of albumin and other circulating compounds can cause astrogliosis, characterized by the upregulation of GFAP (glial fibrillary acidic protein), astrocyte swelling and proliferation (David et al., 2009; Das et al., 2012), and promote extracellular K^+^ accumulation by indirect effects (Ivens et al., 2007; Janigro, 2012), favoring seizural activity by mechanisms described below.

#### Connexins and astrocytes control nutrient supply to the nervous tissue

While astrocytic endfeet contact the BBB endothelium and its associated basement membrane at one side, at the other end, astrocytic processes project toward pre- and postsynaptic neuronal membranes forming the “tripartite synapse” (Allen and Barres, 2009). Here, astrocytes exert homeostatic control over neural network excitability: they provide neurons with energy and substrates for neurotransmission while removing excessive neurotransmitters and K^+^ from the extracellular space by spatial buffering and siphoning. As such, neurons are protected from energy deprivation, large depolarisations and hyperexcitation that would eventually lead to neuronal death.

Astrocytes control blood flow and nutrient (oxygen and glucose) supply to those regions where neuronal activity is high by a process termed functional hyperemia or neurovascular coupling that acts at the level of cerebral arterioles and possibly also pericytes (Zonta et al., 2003 and reviewed in Iadecola and Nedergaard, 2007; Attwell et al., 2010; Petzold and Murthy, 2011). Unlike peripheral arterioles where the vasomotor response is propagated along smooth muscle cells, these do not seem to play a role in the regulation of blood flow in the CNS. Indeed, Xu et al. (2008) have shown that selective astrocyte death abolishes vasodilation despite the continued presence of viable smooth muscle cells (Xu et al., 2008). Although less documented, the vasomodulatory action of astrocytes can also imply a vasoconstriction (Mulligan and MacVicar, 2004; Metea and Newman, 2006; Filosa and Blanco, 2007; Gordon et al., 2008). Candidate astrocyte messengers mediating the vasomotor response include K^+^ as well as gliotransmitters (glutamate, ATP, adenosine, and NO) and arachidonic acid metabolites (reviewed in Mulligan and MacVicar, 2004; Koehler et al., 2006; Iadecola and Nedergaard, 2007; Attwell et al., 2010). A Cx43 mimetic peptide that prevents both HC opening and GJIC, has been shown to prevent neurovascular coupling (Xu et al., 2008); however, it remains to be established exactly how Cx channels contribute to neurovascular coupling. Likely, Cx channels mediate the propagation of ICWs between astrocytes, possibly spreading the vasomotor response. Increased blood flow in local parenchymal capillaries requires for example an upstream vasodilation in arterioles and pial vessels, which might be communicated by Cx-based astrocytic ICWs. ICWs rely on both a direct communication of Ca^2+^ mobilizing messengers mediated by GJs and an paracrine route involving the release of a Ca^2+^ mobilizing messenger in the extracellular space (Leybaert and Sanderson, 2012). Astrocytic Ca^2+^ changes play a key role in neurovascular coupling (Takano et al., 2006), with rises in [Ca^2+^]_*i*_ along the path of the Ca^2+^ wave enabling the release of vasoactive messengers (Zonta et al., 2003; Mulligan and MacVicar, 2004). Additionally, HCs may provide a release pathway for vasoactive substances, yet this remains to be determined. Defective GJIC or HC responses are expected to disable ICWs and neurovascular coupling, resulting in a relative oxygen and glucose shortage during neuronal activity that may lead to convulsions and, on a longer term, to neuronal cell death. Chronically disturbed neurovascular coupling may lead to neurodegeneration (Zlokovic, 2011; Lasta et al., 2013) and this may be a possible mechanism at the basis of the motor deficiencies observed in ODDD patients.

#### Connexins and astrocyte modulation of synaptic transmission

Neuronal action potential firing is always accompanied by an increase of K^+^ levels in the extracellular space. Astrocytes buffer these excessive K^+^ ions through inward rectifier K^+^ channels (Kir4.1) aided by Na^+^/K^+^ pump activity and passive uptake with water through aquaporin 4 channels. Altered activity of both K^+^ channels and Na^+^ channels in the astrocyte PM are considered pro-epileptic (D'Ambrosio, 2004). Subsequently, K^+^ is redistributed over networks of GJ-connected astrocytes (reviewed in Carlen, 2012; Steinhauser and Seifert, 2012). Like K^+^, glutamate, the brain's major excitatory neurotransmitter, is taken up and diluted in those astrocytic networks during synaptic activity, keeping its levels in the synaptic cleft low and sheltering neurons from excitotoxic injury. Inside the astrocytes, glutamate is converted to glutamine that is shuttled back to presynaptic nerve terminals where it is reconverted to glutamate. Disturbed redistribution of glutamate and K^+^ in the astrocytic networks, via GJ loss or abnormal GJ channel gating and permselectivity, may lead to a local accumulation of both substances, paving the way for epileptic seizure activity (reviewed in Pannasch et al., 2011). The swelling of astrocytes may additionally result in a decreased extracellular space volume further increasing ambient concentrations of K^+^ and glutamate sensed by neurons. Reduced expression of Cx43 has been shown to increase expression of glutamate transporters (Unger et al., 2012), likely as a means to compensate the inadequate uptake of glutamate form the extracellular environment; its functional implications are, however, unknown. Also note that the clearance and redistribution of K^+^ is partially maintained in the hippocampus when Cx43 and Cx30 expression is silenced in astrocytes (Wallraff et al., 2006), indicating that mechanisms other than those related to astrocytic Cxs contribute to spatial buffering.

More than just exerting homeostatic control over the extracellular compartment, astrocytes are now considered to also more actively contribute to synaptic transmission, by responding to neuronal activity and releasing gliotransmitters. Individual astrocytes can contact up to 140,000 synapses (Bushong et al., 2002) and express a plethora of neurotransmitter receptors that in most cases trigger an increase in [Ca^2+^]_*i*_. In response to this, they release gliotransmitters that include glutamate, D-serine, ATP, adenosine, and GABA of which neurones on their turn express receptors (Perea and Araque, 2010; Orellana et al., 2011; Pannasch et al., 2012). HCs composed of Cx43 may well-contribute to gliotransmitter release as a [Ca^2+^]_*i*_-controlled diffusive pathway: HCs open with a [Ca^2+^]_*i*_ increase up to 500 nM and close again at higher concentrations (see Cx43 Mutations Located in the Cytoplasmic Loop). Stehberg and co-workers have tested the hypothesis of gliotransmitter release via HC opening by making use of an inhibitory peptide (L2 peptide) that specifically targets Cx43 HCs without inhibiting Cx43 GJs (Ponsaerts et al., 2010). This work, performed *in vivo*, demonstrated that stereotactic injection of this peptide (coupled to a TAT-translocation motif) in the basolateral amygdala, blocked the consolidation of fear memory while addition of a cocktail of putative gliotransmitters rescued the L2-mediated inhibition of fear memory consolidation (Stehberg et al., 2012). Besides being activated by a moderate [Ca^2+^]_*i*_ increase, HCs are also activated by a lowering of extracellular Ca^2+^. Extracellular Ca^2+^ can decrease as a result of neuronal activity and Nedergaard and co-workers have recently demonstrated that this may trigger astrocytic HC opening with consequent ATP release (Torres et al., 2012). The latter was demonstrated to activate inhibitory interneurons that put a brake on excitatory firing (Torres et al., 2012). Additionally, adenosine, derived from ATP degradation, has anticonvulsant effects as it inhibits presynaptic neurotransmitter release (Pascual et al., 2005). Overall, HC activation in astrocytes may thus contribute to gliotransmitter release, but with the evidence presently available, this may lead to excitatory as well as inhibitory signaling.

The contribution of Cx channels to ictogenesis has an ambiguous, two-faced aspect: on the one hand GJs have an anti-convulsive effect with respect to their K^+^ and glutamate buffering capacity, but on the other hand, GJs may act in a pro-convulsive way as well. In this respect, Cx43 gene ablation or pharmacological block of GJs reduced seizure activity while agents that potentiate cell–cell coupling enhanced neuronal bursting (Kohling et al., 2001; Wallraff et al., 2006; Yoon et al., 2010). Mechanistically, neuron-derived glutamate may trigger a [Ca^2+^]_*i*_ increase in astrocytes that further stimulates glutamate release from these cells. The Ca^2+^ signal can propagate to neighboring astrocytes in the form of an intercellular Ca^2+^ wave, modulating groups of remotely located synapses and inducing a hyperexcited [Ca^2+^]_*i*_ state over the entire astrocyte network (Giaume, 2010). Additionally, astrocyte GJs provide an intercellular route for the supply of energy substrates like glucose and lactate that are required for neuronal activity (Rouach et al., 2008). Once the astrocytic distribution of these substrates is compromised, energy delivery may become below demand, causing the accumulation of glutamate and K^+^ that may act in a pro-convulsive manner. Finally, both GJs and HCs have been implicated in the spread of apoptosis between astrocytes (Nodin et al., 2005; Eugenin and Berman, 2007; Decrock et al., 2009) which may well-break ground for epileptic seizures (Briellmann et al., 2002; Willoughby et al., 2003; Kang et al., 2006). With respect to HCs, Cx43 mutations giving a gain of HC function (I31M, G143S, and G138R) are, theoretically, expected to result in increased neuronal cell death, caused by excessively elevated neuronal [Ca^2+^]_*i*_ (reviewed in Bennett et al., 2012). However, up to date, none of these gain-of-function mutants has been associated with epileptic seizures in ODDD patients (Abrams and Scherer, 2012). On the other hand, some mutants that result in a loss of HC function (L90V and I130T) are also associated with epileptic seizure activity which may result from a decrease in ATP release and consequent inhibitory signaling. At this stage, it is not clear what the net effect of an astrocytic HC contribution would be on excitability and ictogenesis but it emerges that the involvement of HCs in ictogenesis is, like for GJs, a double-edged sword. The use of novel tools such as the Gap19 peptide (Wang et al., 2013a) and the L2 peptide (Ponsaerts et al., 2010; Stehberg et al., 2012) may shed more light on the role of HCs in the healthy or pathological brain. In comparison to non-specific Cx channel blockers and peptides like Gap26/Gap27, L2/Gap19 peptides specifically block HCs composed of Cx43 (but not those composed of Cx40 and Panx1) while not blocking GJs.

When looking over the currently available evidence for ODDD-associated epilepsy, seizures have been reported in patients exhibiting mutants that give both dysfunctional HCs and GJs (Table 2); the loss of efficient K^+^ and glutamate buffering and inadequate nutrient supply may thus play a primary role in the appearance of an epileptic phenotype in ODDD, although this requires further research. Epilepsy has thus far not been reported for mutants with a gain of HC function.

**Table 2:** **List of ODDD-linked mutations associated with epileptic seizures and their effect on Cx43 HCs and GJs**.

**Mutant**	**PM presence and plaque formation**	**GJIC**	**HC function**
G2fs	n.a.	n.a.	n.a.
R76S	Normal presence in PM	X	n.a.
L90V	Normal GJ plaques	↓	X
I130T	Increased presence in PM but reduced number of GJ plaques	↓	X

*n.a., no data available*.

*X, absence of detectable GJIC or HC activity*.

#### Connexins and astrocyte-oligodendrocyte interactions involved in axonal myelination

As a last example of the astrocytic contribution to normal CNS function we highlight the role of astrocytes in axonal myelination. Astrocytes themselves do not produce myelin, but they are connected to oligodendrocytes, the brain's myelinating cells, via Cx43/Cx47 GJs (Orthmann-Murphy et al., 2007). Loss-of-function mutations in the Cx47 gene (*GJA12/GJC2*) lie at the basis of Pelizaeus-Merzbacher-like disease (PMLD1), an early onset, progressive dysmyelinating disorder affecting the CNS, and are also known to cause a distinct form of late onset hereditary spastic paraplegia (SPG44). In both cases, Cx47 mutations prevent the formation of functional Cx47/Cx47 and Cx43/Cx47 GJs causing hypomyelinating leukoencephalopathy (Orthmann-Murphy et al., 2007, 2009). The role of astrocyte-oligodendrocyte GJs is unclear but possibly relates to K^+^ shunting between both cell types. Although astrocytes and oligodendrocytes are also connected through Cx30/Cx32 GJs, these do not seem to compensate for the loss of Cx43/Cx47 coupling, probably as a consequence of differences in conductance, gating, and permeability (Orthmann-Murphy et al., 2007). We expect that spasticity observed with ODDD is similarly related to non-functional Cx43/Cx47 GJs caused by mutations in the *GJA1* gene; however, there are, to our knowledge, no records that address astrocyte-oligodendrocyte coupling with ODDD-linked Cx43 mutants. The observation of a severe downregulation of astrocytic Cx43 in demyelinating lesions of patients suffering from Baló's disease, a disorder characterized by astrocytopathy and demyelination (Masaki et al., 2012), may give credit to the hypothesis that aberrant Cx43 function in astrocytes results in anomalous axonal myelination.

Finally, the retention of processed or unprocessed Cx43 protein can cause glial cell death through a process known as the “unfolded protein response” or through ER stress (reviewed by Roussel et al., 2013). The unfolded protein response and ER stress have been implicated in different neurodegenerative diseases, including those with progressive motor dysfunction (Huntington's disease, amyotrophic lateral sclerosis) as observed in ODDD as well.

### Implications of ODDD-linked mutations in CNS cells other than astrocytes

#### BBB-ECs

The role of Cxs, including Cx43, in BBB endothelial cells is only starting to emerge. Endothelial GJs have been implicated in maintenance of the interendothelial junctional complex (Nagasawa et al., 2006) and more recently, we have shown that Ca^2+^ dynamics triggered by low extracellular Ca^2+^ or inflammatory substances were sustained by GJs and HCs and contributed to disturbed BBB function and increased permeability (De Bock et al., 2011, 2012, 2013). Additionally, work from others has indicated that Cx43 HCs contribute to endothelial cell loss during pathologic insults (Danesh-Meyer et al., 2012). The effect of a Cx43 loss-of-function has not been studied at the level of the BBB, although one might expect a dysfunctional barrier due to a disorganisation of the junctions. On the other hand, following the observation that HCs contribute to increasing permeability, those mutants exhibiting increased HC activity are expected to render the BBB leaky to circulating compounds. The implications of such a leaky BBB have been outlined above.

#### Neurons

Cx43 is not expressed in adult neurons but it is prominently present in neuroblast cells where its role in embryonic development of the cortex becomes more and more acknowledged. Radial glial cells, the neuronal stem cells of the embryonic cerebral cortex, originate in the ventricular zone and differentiate into neuronal cells as the neocortex forms. These neuronal cells subsequently migrate to their final destination in the cortical plate. Additionally, radial glia give rise to radial fibers that form a guidance scaffold for neuronal migration. Cx43 can be found in the neuronal progenitors (or neuroblasts) as well as in the glial scaffold. Expression of the Cx43-T154A mutant that is able to make adhesions but is unable to form functional GJ channels in the developing cortex did not abolish the migration of new born neurons, but expression of the Cx43-C61S mutant that lacks docking ability (with maintained HC function) did prevent migration of new neurons. These observations suggested that channel activity does not play a role but Cxs are involved as adhesive contact points between the scaffold and the migrating neurones (Elias et al., 2007). More recently, it was shown that conditional Cx43 knock-out in radial glia disrupts neuronal migration, and this could be rescued by expression of full-length Cx43 but not by expression of CT-truncated Cx43 (removal of the last 125 residues) (Cina et al., 2009). These findings indicate that the C-terminal tail, which is known to interact with scaffolding proteins like ZO-1 and cytoskeletal tubulin (Giepmans et al., 2001), crucially links adhesive Cx43 properties to the cytoskeleton. Cx43 additionally co-localizes with proteins specialized in cell adhesion to neighboring cells or to the extracellular matrix (Nagasawa et al., 2006; Li et al., 2010; Sato et al., 2011). Although both studies suggest that the Cx43 channel pore has no function in neuronal migration, other studies have indicated that Cx43 HCs and GJs play a role in the initiation and propagation of Ca^2+^ signals in and between neuronal precursor cells respectively. These Ca^2+^ signals are likely to play a role in neuronal proliferation. Blocking HCs/GJs reduced neuronal motility, likely by interfering with Rho-GTPase activity, and gave rise to defective neurogenesis (Weissman et al., 2004; Liu et al., 2008, 2010). Cx43 knock-out mice generally do not present with a severely distorted cortex which might be explained by the fact that other Cxs like Cx30 or Cx26 contribute to neuronal migration (Elias et al., 2007), compensating for the loss of Cx43. One mouse strain that lacks Cx43 in embryonic radial glia, termed Shuffler, mimics part of the neuronal phenotype observed in ODDD (gait disturbance and ataxia). The mice exhibit structural abnormalities in the cerebellum, hippocampus and cortex which can explain the neuronal deficits. Importantly, the phenotype of these mice differs from other Cx43 knock-out mice strains in which no neurological phenotype was observed, suggesting that the genetic background is an important determinant of the occurrence of neurological symptoms (Wiencken-Barger et al., 2007). Such effect may apply for humans as well and might account for the rather low prevalence of neurological manifestations in ODDD patients.

#### Microglia

Microglial cells are the resident innate immune effector cells of the CNS and are essential in the primary defense against pathologic insults. Resting amoeboid microglia continuously scan the nervous tissue and rapidly respond to inflammatory molecules, pathogens or tissue injury by transforming into ramified cells that exhibit a high rate of proliferation and migration (reviewed in Kettenmann et al., 2011). Their activation involves the release of pro-inflammatory cytokines, free radicals and glutamate (Candelario-Jalil et al., 2007; Sumi et al., 2010; Takeuchi et al., 2011) which are all generally believed to be neurotoxic. Indeed, microglial activation is observed in many neurodegenerative diseases including Alzheimer's disease, Parkinson's disease, Huntington's disease, and amyotrophic lateral sclerosis (Orellana et al., 2009) as well as in epilepsy (reviewed in Mirrione and Tsirka, 2011; Vezzani et al., 2012). In addition to direct neurotoxic effects, cytokines and other molecules released by microglia may inhibit GJIC between astrocytes (Meme et al., 2006) while stimulating HCs (Retamal et al., 2007), or activate BBB endothelial cells (Orellana et al., 2009), further contributing to neurodegeneration and epilepsy via mechanisms described above. However, in certain scenarios, microglia may also contribute to CNS repair through scavenging of reactive oxygen species, the release of neurotrophic factors and the removal of neurotoxic substances (Rouach et al., 2004, reviewed in Mika and Prochnow, 2012; Aguzzi et al., 2013). The role of Cx43 in the CNS' immune cells is uncertain. Whereas some groups find no indication for the presence of microglial Cx43 in the resting or ramified state (Dobrenis et al., 2005; Theodoric et al., 2012), others do report an increase in microglial Cx43 in inflammatory conditions (Eugenin et al., 2001; Garg et al., 2005; Shaikh et al., 2012). HCs have been implicated in glutamate release which could contribute to neuronal/astrocyte cell death and epilepsy (Ye et al., 2003; Takeuchi et al., 2008). In addition, dye coupling between microglial cells has been observed upon exposure to inflammatory conditions (Eugenin et al., 2001; Martinez et al., 2002). The functional implications of this activation-dependent microglial coupling remain unclear. Similar as in peripheral immune cells where Cx43 levels increase during inflammation, GJs could be involved in the cell–cell transfer of immunogens, rendering antigen cross-presentation more efficient (Neijssen et al., 2005). Altogether, altered Cx43 expression/channel function could disturb the proper microglial response to inflammation, failing in the defense against neurodegenerative mediators but contributing to neuronal bursting activity.

## Conclusions

ODDD manifests as a pleiotropic disease with patients exhibiting both morphological and functional deficiencies caused by mutations in the widespread *GJA1* gene. The *GJA1* gene product Cx43 plays a leading role in CNS physiology and it thus comes with no surprise that neurological symptoms are included in the still expanding list of ODDD features. Analysis of the different mutants demonstrates that they can either alter insertion of Cx43 in the PM, GJ channel formation or HC/GJ channel gating. All CNS cells expressing aberrant Cx43 are potential contributors to nervous tissue dysfunction or damage, with a major involvement of astrocytes as the prime cell type expressing Cx43. In this paper we gave an overview of those mutants associated with nervous tissue dysfunctioning along with their outcome on Cx43 function. The information currently available on the possible effects of ODDD-linked mutations on Cx43 expression/channel function is impressive, as can be appreciated from Table 1. However, there is still a paucity of reports that document and analyze the mechanism by which the mutants give the neurological phenotype in a detailed manner. Thus, it remains difficult to present a clear genotype/phenotype correlation. None of the mutants identified thus far have been characterized for their specific effect on the function of astrocytes or other cells of the neurogliovascular unit, and therefore, genotype/phenotype linkage still remains in the realm of speculation. ODDD is, with a few exceptions, subject to an autosomal dominant inheritance pattern but unfortunately, thus far, there is no clear hypothesis that explains this pattern. Having one dysfunctional allele may cause a CNS phenotype in ODDD while in homozygous astrocyte-specific Cx43 knock-out animals no severe alterations are observed (Theis et al., 2003). This observation may plead in favor for a dominant negative effect of mutant Cx43 on co-expressed WT Cx43 protein. Most of the Cx43 mutants exhibit dominant negative effects, but a comparison of dominant negative mutants and neurological phenotypes is unfortunately inconclusive. The dominant negative effect of Cx43 mutants does not guarantee neurological findings (see for instance L11P and S18P) while mutants that do not exert a dominant effect may give a severe neurological phenotype (I130T). Trans-dominant negative effects of the mutants on other co-expressed Cxs are another likely explanation. Such effects have for instance been used to explain the dominant expression pattern of syndromic deafness caused by *GJB2* (Cx26) mutations (reviewed in Laird, 2008). On the other hand, loss-of-function mutations of Cx26 and Cx30 have thus far not been associated with neuropathology (Abrams and Scherer, 2012). In the heart, Cx43-R33X mutants exert trans-dominant effects on Cx37 and Cx40 (Huang et al., 2013), whereas G138R does not (Dobrowolski et al., 2008). Unfortunately, we are not aware of any studies tackling this question with regards to the CNS. The (trans-)dominant effect of Cx43 mutants may additionally be cell type specific as the mouse G60S mutant has dominant negative effects on WT Cx43 in ovarian granulosa cells (Flenniken et al., 2005) but not in astrocytes (Wasseff et al., 2010). Finally, as illustrated in Table 2, epilepsy seems to be associated with loss of function ODDD mutants. We carefully checked this for motor deficiencies (Table 3) and found that a motor phenotype is always associated with mutants that give a loss or reduction of GJs, while the mutant's effects on HC function are variable. This generates a number of interesting working hypotheses that are open to be tested, hopefully strengthening our understanding of how ODDD mutants lead to channelopathy, CNS cell dysfunction and neurodegeneration.

**Table 3:** **List of ODDD mutations linked to upper motor neuron dysfunction, tremor, gait disturbances, and spasticity, indicating neurodegeneration**.

**Mutant**	**PM presence and plaque formation**	**GJIC**	**HC function**
D3N	Normal number of plaques	↓	n.a.
Y17S	Reduced presence in PM with reduced number of GJ plaques	X	X
G21R	Reduced number of GJ plaques	X	X
K23T	Normal GJ plaques	n.a.	n.a.
R33X	Not present in PM	X	X
A40V	Reduced number of GJ plaques	X	X
L90V	Normal GJ plaques	↓	X
H95R	n.a.	n.a.	n.a.
L106P	n.a.	n.a.	n.a.
L113P	n.a.	n.a.	n.a.
I130T	Increased presence in PM but reduced number of GJ plaques	↓	X
K134E/N	Reduced number of GJ plaques	↓	X
G138R	Increased presence in PM with normal number of GJ plaques	X	↑
T154A/N	Normal number of GJ plaques	↓	n.a.
V216L	Reduced number of GJ plaques	↓	n.a.

*n.a., no data available*.

*X, absence of detectable GJIC or HC activity*.

### Conflict of interest statement

The authors declare that the research was conducted in the absence of any commercial or financial relationships that could be construed as a potential conflict of interest.

## Footnotes

^1^http://www.orpha.net
